# Trait Shifts and Vertical Redistribution Enable Nematode-Trapping Fungi to Adapt to Fire Disturbance

**DOI:** 10.3390/jof12080611

**Published:** 2026-08-14

**Authors:** Rong She, Xin Zhang, Cai-Lian Yuan, Yu Sun, Xiao-Yan Yang, Wen Xiao

**Affiliations:** 1 Institute of Eastern-Himalaya Biodiversity Research, Dali University, Dali 671003, China; sher@eastern-himalaya.cn (R.S.);; 2 Collaborative Innovation Center for Biodiversity and Conservation in the Three Parallel Rivers Region of China, Dali 671003, China; 3 The Provincial Innovation Team of Biodiversity Conservation and Utility of the Three Parallel Rivers Region from Dali University, Dali 671003, China

**Keywords:** fire disturbance, functional traits, nematode-trapping fungi, trait shifts, vertical redistribution, microbial resilience

## Abstract

Fire rapidly reshapes soil environments, yet direct experimental evidence of how soil microorganisms respond through both functional trait adjustment and spatial redistribution remains limited. Here, we used nematode-trapping fungi (NTF) as a model system to test whether their response to fire is mediated by coordinated changes in functional traits and vertical redistribution. We first compared strains isolated from naturally burned habitats with those from adjacent unburned habitats and identified consistent trait shifts; we then tracked NTF dynamics in artificial soil profiles exposed to simulated fire. Strains from burned habitats produced 53–1114% more trapping structures and exhibited bait-based predation efficiencies 9–41% higher than those from unburned habitats. Conidia of fire-adapted strains were smaller, with reductions in length and width of 0.3–8.0 µm and 0.1–3.4 µm, respectively. Hyphal growth rates increased significantly in *Dactylellina* and *Drechslerella* but declined overall in *Arthrobotrys*. In the soil-profile microcosm experiment, simulated fire completely eliminated *Arthrobotrys* from the topsoil (0–5 cm), whereas *Dactylellina* and *Drechslerella* from deeper layers (10–20 cm) were subsequently detected in upper layers, indicating upward redistribution and recolonization within 7–56 days. Together, these results reveal two complementary mechanisms underlying NTF adaptation to fire: individual-level trait shifts, which likely reflect the reallocation of resources from reproductive structures to predatory traps and hyphal expansion, and community-scale vertical redistribution, which enables recolonization via deep-soil source populations. Our work provides experimental evidence that soil microorganisms cope with abrupt fire disturbance through coordinated trait shifts and vertical spatial redistribution, offering mechanistic insights that may support trait-oriented restoration management of post-fire soil ecosystems.

## 1. Introduction

Soil microorganisms are central to carbon and nitrogen cycling and underpin the stability and resilience of terrestrial ecosystems [1]. Under ongoing climate warming, wildfire frequency and intensity are projected to increase by 20–50% in the coming decades, making fire a dominant disturbance in forest and grassland soils [2,3]. In contrast to gradual environmental changes such as warming or drought, fire is an abrupt, high-intensity disturbance that can rapidly reshape soil environments through intense heating and resource redistribution [4,5].

Fire can raise topsoil temperatures above 200 °C, causing extensive microbial mortality. In contrast, deeper soil layers are often less affected because heat declines rapidly with depth, creating a pronounced vertical environmental gradient [6,7]. Fire also reshapes soil nutrient conditions through organic matter combustion and nitrogen volatilization, often intensifying short-term nitrogen limitation [8,9]. This combination of severe disturbance and strong spatial heterogeneity makes post-fire soils a powerful natural system for studying how microorganisms respond to rapid environmental change.

Organisms generally respond to environmental change through three mechanisms: phenotypic plasticity, dispersal, and genetic adaptation [10,11,12]. For soil microorganisms, however, strong spatial constraints mean that genetic adaptation is unlikely to dominate recovery during the early post-fire period, which unfolds over weeks to months [13,14]. Rapid trait shifts and spatial movement, particularly vertical redistribution, are therefore more likely to govern immediate microbial responses to fire [15,16,17]. Here, we use “trait shifts” to refer to observable differences in functional traits associated with contrasting fire histories, without implying a specific underlying mechanism. These shifts may arise from phenotypic plasticity, genetically based differentiation, or environmental filtering; distinguishing among these possibilities requires experimental designs that lie beyond the scope of the present study. Yet most studies have focused on shifts in community composition or functional genes, with little direct experimental evidence linking changes in individual functional traits to spatial redistribution processes [18].

Nematode-trapping fungi (NTF) provide an ideal model system for testing these ideas. These fungi can switch flexibly between saprotrophic and predatory lifestyles, and the formation of trapping structures such as adhesive networks and constricting rings can be induced by nitrogen limitation or high nematode density. Key functional traits, including trapping-structure production, predation efficiency, conidial size, and hyphal growth rate, can be quantified directly and are closely related to resource-acquisition strategy and spatial expansion [19,20,21,22]. Previous studies suggest that post-fire NTF communities often shift from more saprotrophic taxa, such as *Arthrobotrys*, to more predatory taxa, such as *Dactylellina* and *Drechslerella*, the latter of which tend to occur in deeper soil layers under natural conditions [23,24,25]. However, direct evidence of whether this community shift is driven by trait adjustments, vertical redistribution, or a combination of both remains lacking.

Here, we combined comparative trait analyses of NTF strains isolated from naturally burned and adjacent unburned habitats with an artificial soil-profile experiment to explore how NTF respond to fire through trait adjustments and spatial redistribution. We tested two hypotheses: (1) NTF strains from naturally burned habitats exhibit functional traits consistent with post-fire resource acquisition and colonization; and (2) NTF in deeper soil layers can redistribute upward to replenish and rebuild topsoil communities. Although the trait comparison and soil-profile detection were conducted on different sets of strains, making it impossible to directly link individual trait changes to vertical redistribution within the same populations, integrating these two lines of evidence provides complementary insights into the potential mechanisms underlying post-fire microbial recovery. By integrating individual-level trait responses with community-level spatiotemporal dynamics, this study aims to provide a more mechanistic understanding of how soil microorganisms respond to rapid environmental change.

## 2. Materials and Methods

### 2.1. Trait Shifts Experiment

#### 2.1.1. Fungal Strains

We selected three common genera of nematode-trapping fungi (NTF): *Arthrobotrys*, *Dactylellina*, and *Drechslerella*. For each genus, three representative species were included. To minimize potential confounding between habitat and site effects, strains were obtained from three independent wildfire sites, each paired with an adjacent unburned site of similar vegetation and soil type located <500 m away. For each species, one strain per habitat was isolated from each paired site, yielding three strains per species per habitat from geographically distinct locations. Strains were isolated via the nematode-bait sprinkle plate method, and single-conidium transfer was performed to establish pure cultures. In total, 54 strains were used (3 genera × 3 species × 3 strains × 2 habitat types; see Supplementary Table S1).

All strains were identified using both morphological and molecular approaches (ITS rDNA sequencing) and are maintained in the Microbial Germplasm Resource Collection of the Institute of Eastern Himalaya Biodiversity Research, Dali University. These strains had been previously isolated and identified as part of our ongoing NTF biodiversity survey. Before the experiment, each strain was revived from stock culture and re-confirmed by ITS rDNA sequencing to ensure its identity. Voucher specimens and sequence data are available upon request.

#### 2.1.2. Strain Revival and Propagation

Strains were revived by transferring mycelium from storage tubes onto corn meal agar (CMA) plates using a sterile toothpick and incubating them at 26.5 °C following Zhang and Hyde [26].

#### 2.1.3. Measurement of Conidial Size

Conidial morphology was measured using a modified microscopic method [27]. Each strain was inoculated onto CMA and induced to produce conidia on coverslips. After incubation, coverslips were removed, mounted on slides, and examined under an Olympus BX53 microscope (Olympus Corporation, Tokyo, Japan). Conidial length and width were measured (Supplementary Figure S1A,B).

For each strain, 300 mature conidia were measured, resulting in 16,200 measured conidia across all 54 strains.

#### 2.1.4. Measurement of Hyphal Growth Rate

Hyphal growth rate was quantified as colony diameter after a fixed incubation period [28]. Activated strains were first transferred to fresh CMA plates by single-conidium isolation and incubated at 26.5 °C until conidial production peaked (after 7 days). Conidia were then washed off with 0.9% sterile saline to prepare a suspension adjusted to approximately 200 conidia per 100 µL after counting with a hemocytometer. A 10 µL aliquot of the suspension (approximately 20 conidia) was inoculated at the center of a 90-mm potato dextrose agar (PDA) plate and incubated at 26.5 °C for 7 days, after which colony diameter was measured (Supplementary Figure S1C). Each strain was assayed with five replicates.

#### 2.1.5. Measurement of Trapping-Structure Production and Predation Efficiency

Trapping-structure production and predation efficiency were assessed using a custom-built observation device (patent number: CN201621242750; Supplementary Figure S2). Each strain was tested with five replicates.

On each 60-mm CMA plate, a circular observation chamber (2 cm in diameter) and four conidial inoculation points were prepared. The bottom of the chamber was filled with 200 µL of CMA medium at approximately 50 °C. After solidification, 10 µL of NTF conidial suspension (prepared as above, approximately 20 conidia) was added to each of the four inoculation points; the conidial suspension was prepared as described above for the hyphal growth assay. Plates were incubated at 26.5 °C until the observation chamber was fully colonized by hyphae, after which 20 µL of nematode suspension containing approximately 500 individuals of the free-living bacterial-feeding nematode *Panagrellus redivivus* was added.

Formation of trapping structures (see Supplementary Figure S3 for trap morphologies; the figure note specifies which fungal genus produces each trap type) was observed every 12 h for 72 h under a T-vision microscope (T-Vision Co., Ltd., Guangzhou, China), and the number of dead nematodes was recorded. Predation efficiency was calculated using a corrected formula to account for natural mortality in the control treatment [29], where R is the corrected predation efficiency, X is the number of nematodes preyed upon in the treatment, Y1 is the number of freely moving nematodes in the treatment, Z is the number of naturally dead nematodes in the control, and Y2 is the number of freely moving nematodes in the control.

$$R=\frac{X}{X+Y1}\times 100\%-\frac{Z}{Z+Y2}\times 100\%$$

### 2.2. Vertical Migration Experiment

#### 2.2.1. Experimental Design

To simulate natural vertical distributions in soil profiles, the topsoil-dominant species *Arthrobotrys oligospora* (strain DL219) from unburned habitats was inoculated into the 0–5 cm layer, whereas the deep-soil-dominant species *Dactylellina ellipsospora* (strain DL251) and *Drechslerella dactyloides* (strain LP155) from unburned habitats were inoculated into the 10–20 cm layer. The morphological characteristics of the three nematode-trapping fungal strains used in the experiments are shown in Supplementary Figure S3. *A. oligospora* is characterized by its conidia and the formation of adhesive networks (Supplementary Figure S3A); *Dac. Ellipsospora* produces adhesive knobs (Supplementary Figure S3B); and *Dre. dactyloides* forms constricting rings to capture nematodes (Supplementary Figure S3C). These images are adapted from the monograph *Nematode-trapping fungi*, edited by Ke-Qin Zhang and Kevin D. Hyde [26]. We define the term “vertical redistribution” operationally to describe the detection of a species in soil horizons above or below its initial inoculation depth over time, regardless of the underlying mechanisms (hyphal outgrowth, passive water transport, etc.).

Three treatment groups were established, each with three replicates: Group A: natural vertical distribution + fire treatment; Group B: natural vertical distribution + additional deep-soil inoculation of *A. oligospora* + fire treatment; and Group C: natural vertical distribution + no fire treatment (control).

#### 2.2.2. Soil Preparation

Soil was collected from the 0–20 cm layer of a pine forest on Mt. Cangshan, Dali, Yunnan, China. After removing twigs, litter, and stones, soil was homogenized and sterilized using tyndallization to eliminate all biotic components. Specifically, soil was autoclaved at 121 °C for 30 min, incubated at 37 °C for 1 h, and this cycle was repeated three times. Sterilized soil was then stored in sterile containers until use.

#### 2.2.3. Construction of Soil Profiles

Sterilized soil was placed into cement pots (40 × 25 × 25 cm), the inner walls of which had been flame-sterilized before use. Fungal inocula were evenly mixed evenly into the designated soil layers according to the design described above. After assembly, each pot was sealed to prevent external contamination and left under field conditions for 10 days to allow the system to stabilize.

To assemble the fungal communities, activated fungus was inoculated onto CMA plates and cultured until the mycelium fully covered the plate surface, forming a mycelial lawn. Then, a sterile cork borer (6 mm in diameter) was used to excise the mycelial lawn together with the underlying agar, obtaining mycelial plugs (Supplementary Figure S4). Thirty plugs of each species were mixed into the corresponding sterile soil layer with a sterile glass rod before the soil was placed into the pot (Supplementary Figure S5). Soil for each pot was prepared separately. After filling, the top opening and bottom drainage holes of each pot were immediately sealed with cellophane to prevent the invasion by external microorganisms and to limited excessive rainwater infiltration, thereby reducing the potential for water-driven passive movement of fungal propagules (Supplementary Figure S6). Soil moisture was not actively monitored during the experimental period. The pots were then buried in farmland soil at the Dali University teaching field station, with the upper rim of each pot kept 5 cm above the ground surface to prevent rainwater infiltration. After 10 days of stabilization, Groups A and B were subjected to simulated fire treatment.

#### 2.2.4. Simulated Fire Treatment

Fire disturbance was simulated by heating the soil surface with a blowtorch positioned 10 cm above the soil for 90 min [30] to simulate a moderate to high severity surface fire in the pine forests of Mt. Cangshan, Yunnan, the region from which the soil were collected. In this ecosystem, surface fires typically consume accumulated pine needle litter and can produce soil surface temperatures above 500 °C for brief periods, with temperatures at 2 cm depth often exceeding 200 °C. The 474 °C recorded at 2 cm in our study is consistent with reported peak temperatures measured at similar depths during experimental fires in pine forests, particularly when a layer of dry litter is present on the soil surface.

To monitor temperature dynamics during heating, an additional blank pot of the same size and material was filled with sterile soil and fitted with thermocouples (Omega Engineering Inc., Norwalk, CT, USA) at depths of 2, 5, 10, 15, and 20 cm below the surface. Thermocouples were inserted horizontally into the soil through pre-drilled holes in the pot wall, and the sensing tips were fully embedded in the soil matrix at the specified depths, ensuring that recorded temperatures reflected actual soil heating rather than direct flame exposure. Temperature monitoring was conducted in this single dedicated pot to avoid disturbing the experimental units; therefore, the thermal profile is descriptive and was used to confirm the presence of a steep gradient rather than for statistical comparisons. The pot was heated under the same conditions, and temperatures were recorded every 10 min until they returned to pre-treatment levels. Full temperature–time curves are provided in Supplementary Figure S7.

#### 2.2.5. Soil Sampling

Soil samples were collected before fire treatment, immediately after the soil temperature returned to pre-treatment levels, and then once per week thereafter until the NTF community in the 0–5 cm layer had stabilized, yielding a total of 16 sampling time points. At each time point, approximately 5 g of soil was collected from each of the 0–5, 5–10, and 10–20 cm layers using a custom-made soil sampler. After sampling, each hole was immediately plugged with a sterile wooden rod of the same diameter, wrapped in aluminum foil, to prevent the sampling hole from serving as an artificial channel for fungal movement and to maintain the integrity of the soil profile. Samples were returned immediately to the laboratory for NTF detection.

In total, 432 samples were collected (3 groups × 3 replicates per group × 3 soil layers per replicate × 16 time points).

#### 2.2.6. Detection of NTF in Soil Samples

For each soil sample, 1 g of soil was evenly distributed onto a 90-mm CMA plate at each of five positions (i.e., 5 g of soil per plate). Three plates were prepared per sample, corresponding to 15 g of soil in total. Approximately 5000 free-living nematodes (*P. redivivus*) were added to each plate as bait to induce NTF germination. Plates were incubated at 20 °C for 4 weeks, after which NTF growth was examined under a stereomicroscope, and species were identified based on conidial morphology. Because this baiting method detects only viable, culturable, and inducible fungi, all reported occurrence data represent bait-based detection rather than direct in situ abundance or quantitative distribution.

All NTF strains used in this study had been previously identified using both morphological characters and ITS rDNA sequencing, and their identities were confirmed before the experiment began. The three genera differed clearly and unmistakably in conidial morphology (Supplementary Figure S3): conidia of *Arthrobotrys* were fusiform, those of *Dactylellina* were long-rod-shaped, and those of *Drechslerella* were lemon-shaped. These morphological characters are strain-specific and non-overlapping, allowing unequivocal identification under a stereomicroscope. Moreover, the experiment was conducted in sealed pots containing sterilized soil, with cellophane barriers preventing the entry of external microorganisms; therefore, any NTF detected during the experiment could only originate from the artificially introduced strains, eliminating the possibility of confusion with native congeneric species. Under these isolated conditions, the three species could be reliably distinguished by conidial morphology alone, and additional molecular confirmation was not essential for determining the presence or absence of the introduced species in each soil layer. The reliability of this method was confirmed in a preliminary validation experiment, in which known pure strains mixed into sterile soil were detected with 100% success using the same protocol.

### 2.3. Data Analysis

All analyses were conducted in R version 4.5.3. Data organization was performed using the packages tidyverse v2.0.0, readr v2.1.5 and janitor v2.2.0. Linear mixed-effects models were fitted using lmer in the lmerTest package v3.1-3, generalized linear mixed-effects models were fitted using glmmTMB v1.1.9, post hoc comparisons were conducted using emmeans v1.10.1, model diagnostics were assessed using performance v0.10.8 and DHARMa v0.4.6, and model summaries were exported using broom.mixed v0.2.9.6.

For the trait comparison experiment, conidial traits were analyzed using strain-level summary data, whereas hyphal growth rate, trapping-structure production, and predation efficiency were analyzed using replicate-level datasets. For conidial traits, each strain-level observation was represented by the mean of 300 mature conidia. Conidial length and width were analyzed separately using linear mixed-effects models, with habitat origin (burned vs. unburned), genus, and their interaction as fixed effects, and species identity as a random effect. This approach avoided pseudoreplication arising from treating individual conidia from the same strain as independent observations. Hyphal growth rate, quantified as colony diameter after 7 days of incubation, was analyzed using a linear mixed-effects model with habitat origin, genus, and their interaction as fixed effects, and strain nested within species as a random effect. Because trapping-structure production was count data, it was analyzed using a generalized linear mixed-effects model with a negative binomial error distribution (NB2). Habitat origin, genus, and their interaction were treated as fixed effects, and strain nested within species was included as a random effect. Predation efficiency was recorded as a percentage; prior to analysis, it was converted to a proportion and logit-transformed, and then analyzed using a linear mixed-effects model with habitat origin, genus, and their interaction as fixed effects, and strain nested within species as a random effect. For all mixed models, the significance of fixed effects was evaluated using Type III analysis of variance. When significant effects were detected, pairwise comparisons between burned and unburned habitat origins were performed within each genus using estimated marginal means with Tukey’s honestly significant difference adjustment for multiple comparisons. Full fixed-effect model outputs are provided in Supplementary Table S2, and Tukey-adjusted pairwise contrasts for all traits are provided in Supplementary Table S3.

To generate species-level summary tables, mean values and standard deviations were calculated separately for burned and unburned habitat origins. These species-level comparisons were used for tabular presentation of trait differences, whereas inference regarding overall habitat and genus effects was based on the mixed-model framework. Independent-samples t-tests were conducted on strain-level means (*n* = 3 strains per habitat per species).

For the vertical redistribution experiment, raw data were recorded at the level of treatment, sampling day, species, soil depth, pot, and bait plate. At each treatment × day × depth combination, three pots were sampled and three bait plates were established per pot, giving nine plates in total. Final occurrence was scored qualitatively: for a given treatment, sampling day, species, and soil layer, a species was classified as “detected” when at least one of the nine bait plates was positive, and as “undetected” only when all nine plates were negative. The number of positive plates out of nine was also recorded (Supplementary Table S1 positive plates). Based on the detection criterion, detection–nondetection matrices were constructed across treatments, sampling times, and soil depths, and used to generate time-depth distribution plots.

To summarize redistribution dynamics, two descriptive metrics were further extracted: first detection time, defined as the first sampling day on which a species was detected in the 5–10 cm or 0–5 cm layer, and topsoil recolonization, defined as whether a species reappeared in the 0–5 cm layer during the observation period. Because the detection data showed strong structural zeros and qualitative threshold-like transitions, and because some generalized linear mixed models exhibited convergence and separation problems, the vertical redistribution component was presented primarily using qualitative spatiotemporal patterns and descriptive redistribution metrics rather than model-based statistical inference. Conclusions drawn from these data are therefore descriptive.

All tests were two-tailed, and statistical significance was assessed at α = 0.05.

## 3. Results

### 3.1. Trait Differences Between Strains from Burned and Unburned Habitats

We compared NTF strains isolated from naturally burned and adjacent unburned habitats with respect to conidial size, hyphal growth rate, and predation-related traits across three genera, nine species, and 54 strains.

#### 3.1.1. Conidial Size

**Figure 1:**
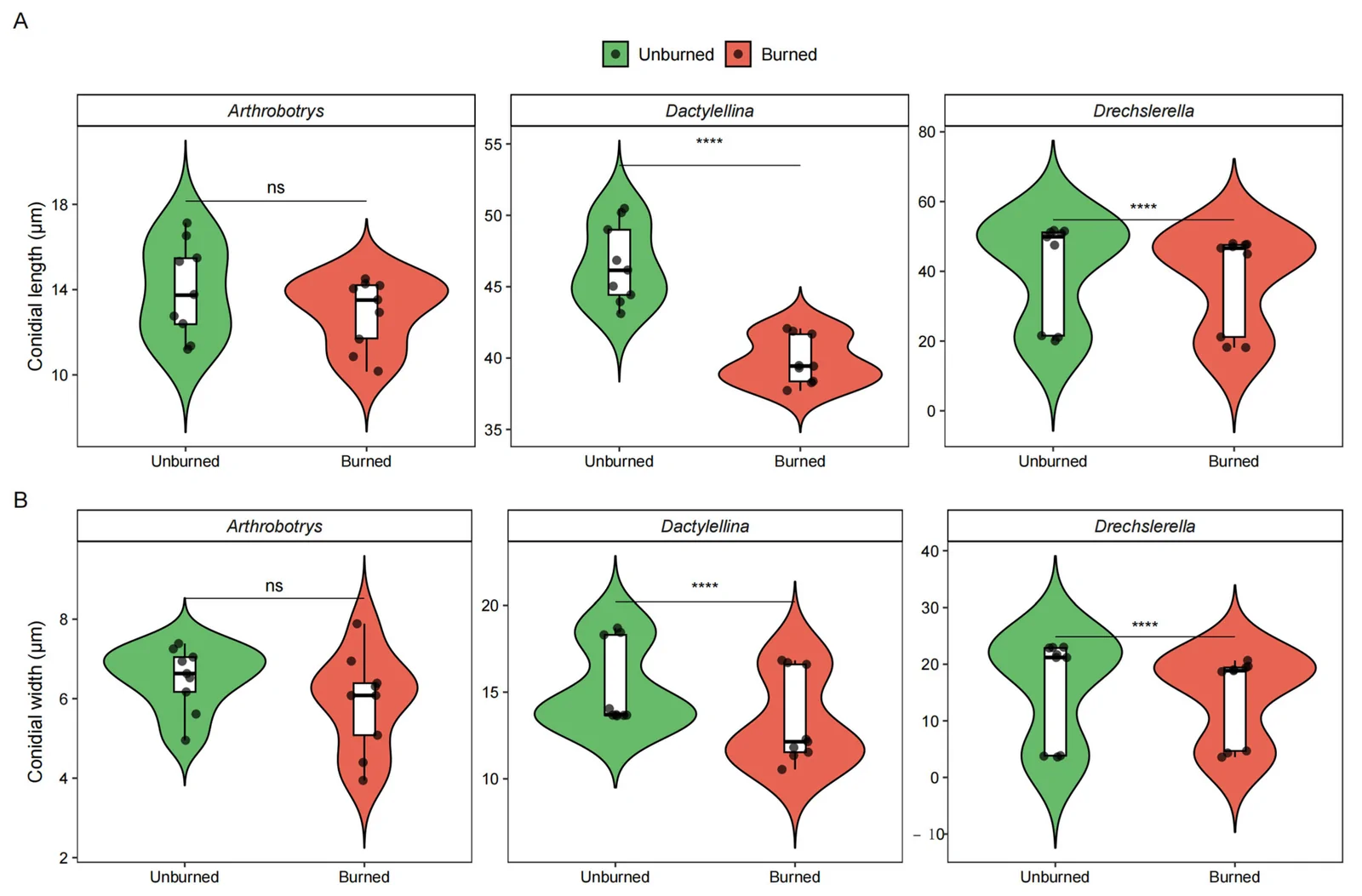
Conidial length (**A**) and width (**B**) of nematode-trapping fungi isolated from naturally burned and adjacent unburned habitats. Violin plots display strain-level mean values for *Arthrobotrys*, *Dactylellina* and *Drechslerella*. Green and red indicate strains from naturally unburned and burned habitats, respectively. Asterisks indicate significant differences between habitat origins within each genus based on Tukey-adjusted mixed-model contrasts; ****: *p* < 0.0001.

Habitat origin exerted genus-specific effects on conidial length and width (habitat × genus interaction: length, *p* < 0.001; width, *p* = 0.015). Linear mixed-model post-hoc contrasts (Tukey’s HSD) revealed that *Dactylellina* and *Drechslerella* strains from burned habitats produced significantly smaller conidia than their conspecifics from unburned sites (all *p* < 0.0001). For *Arthrobotrys*, fire-induced reductions in spore dimensions were weak and marginally non-significant (length, *p* = 0.055; width, *p* = 0.084; Figure 1, Table 1). Tukey-adjusted pairwise contrasts for all traits are provided in Supplementary Table S3.

Species-level *t*-test results were consistent with genus-level mixed-model outputs. Within all three *Dactylellina* species, conidial length was significantly reduced in burned isolates (*Dac. Drechsleri*, *p* = 0.002; *Dac. Ellipsospora*, *p* = 0.006; *Dac. Gephyropaga*, *p* = 0.002); width decreased significantly for *Dac. ellipsospora* and *Dac. gephyropaga* (*p* ≤ 0.003), while *Dac. drechsleri* showed a marginal trend (*p* = 0.055). Among *Drechslerella, D. aphrobrocha* had shorter and narrower conidia (length, *p* = 0.003, width, *p* = 0.028); *D. coelobrocha* exhibited reduced width only (*p* = 0.001), and *D. dactyloides* showed no significant shifts in conidial length (*p* = 0.226), although conidial width was significantly reduced (*p* = 0.001). For *Arthrobotrys*, only *A. musiformis* displayed marginally shorter conidia (*p* = 0.048), whereas *A. conoides* and *A. oligospora* showed no significant size differences between habitat groups (length, *p* = 0.742/0.281; width, *p* = 0.084/0.865). The consistent interspecific trait trends within each genus, alongside divergent genus-level responses, demonstrate that fire history rather than species identity is the primary driver of conidial size variation.

Discrepancies between genus-wide mixed models and species-level independent *t*-tests stem from their distinct statistical frameworks: mixed models incorporated species as a random factor to produce conservative genus-level inferences, while *t*-tests compared only strain mean values for tabular visualization. All core biological conclusions rely on mixed-model outputs, while species-level t-values results serve only supplementary descriptive purposes.

#### 3.1.2. Hyphal Growth Rate

**Figure 2:**
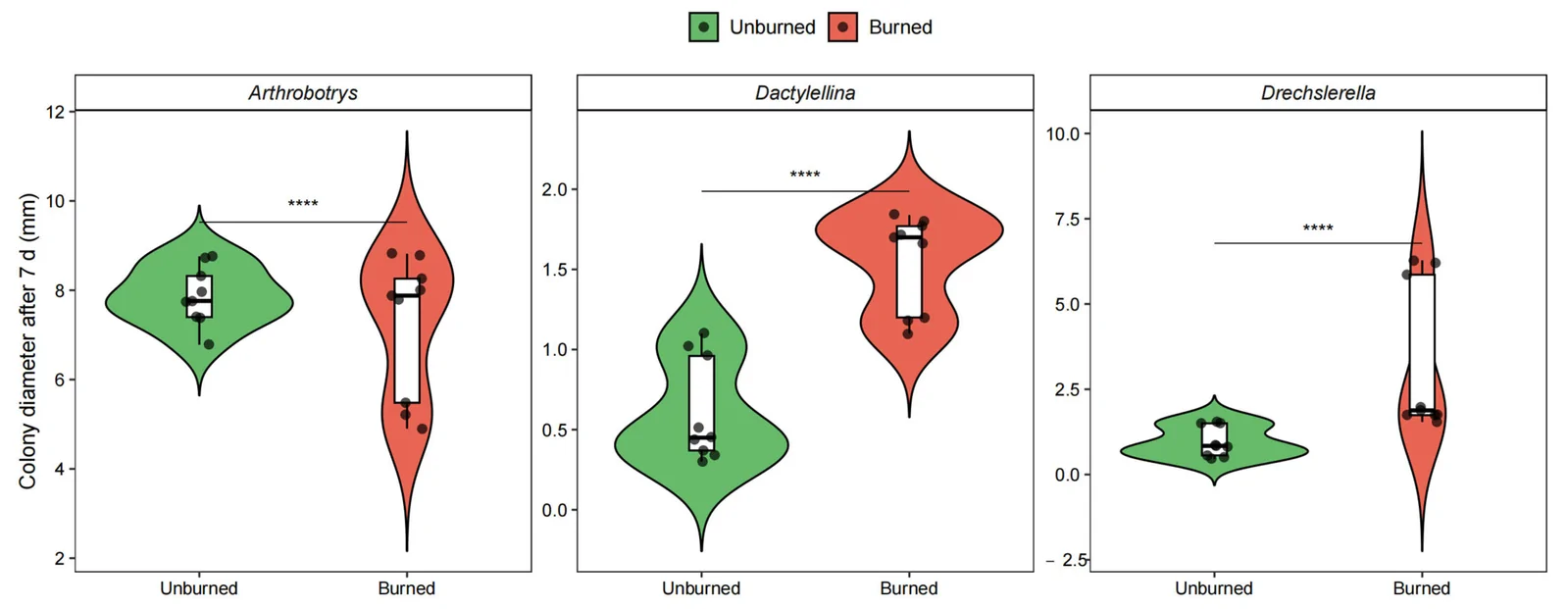
Colony diameter after 7 days of incubation for nematode-trapping fungi isolated from naturally burned and adjacent unburned habitats. Violin plots show strain-level means for the three genera (*Arthrobotrys*, *Dactylellina*, and *Drechslerella*). Green and red indicate strains from unburned and burned habitats, respectively. Asterisks indicate significant differences between habitat origins within each genus based on Tukey-adjusted mixed-model contrasts; ****: *p* < 0.0001.

Hyphal growth rate differed strongly between habitat origins, and the direction of the response depended on genus. Linear mixed-effects models showed significant effects of habitat origin, genus, and their interaction (all *p* < 0.001; Figure 2, Table 2).

Strains of *Arthrobotrys* from naturally burned habitat had smaller colony diameters than those from unburned strains (*p* < 0.0001, Tukey-adjusted), whereas burned-habitat strains of *Dactylellina* and *Drechslerella* had larger colony diameters (both *p* < 0.0001, Turkey-adjusted). Thus, hyphal growth declined overall in *Arthrobotrys* but increased in *Dactylellina* and *Drechslerella*.

#### 3.1.3. Trapping-Structure Production and Predation Efficiency

Predation-related traits increased consistently in strains from burned habitats. For trapping-structure production, the negative binomial generalized linear mixed-effects model showed significant effects of habitat origin, genus, and their interaction (all *p* < 0.001). For predation efficiency, the logit-transformed linear mixed-effects model likewise detected significant effects of habitat origin (*p* < 0.001), genus (*p* = 0.027), and their interaction (*p* < 0.001).

**Figure 3:**
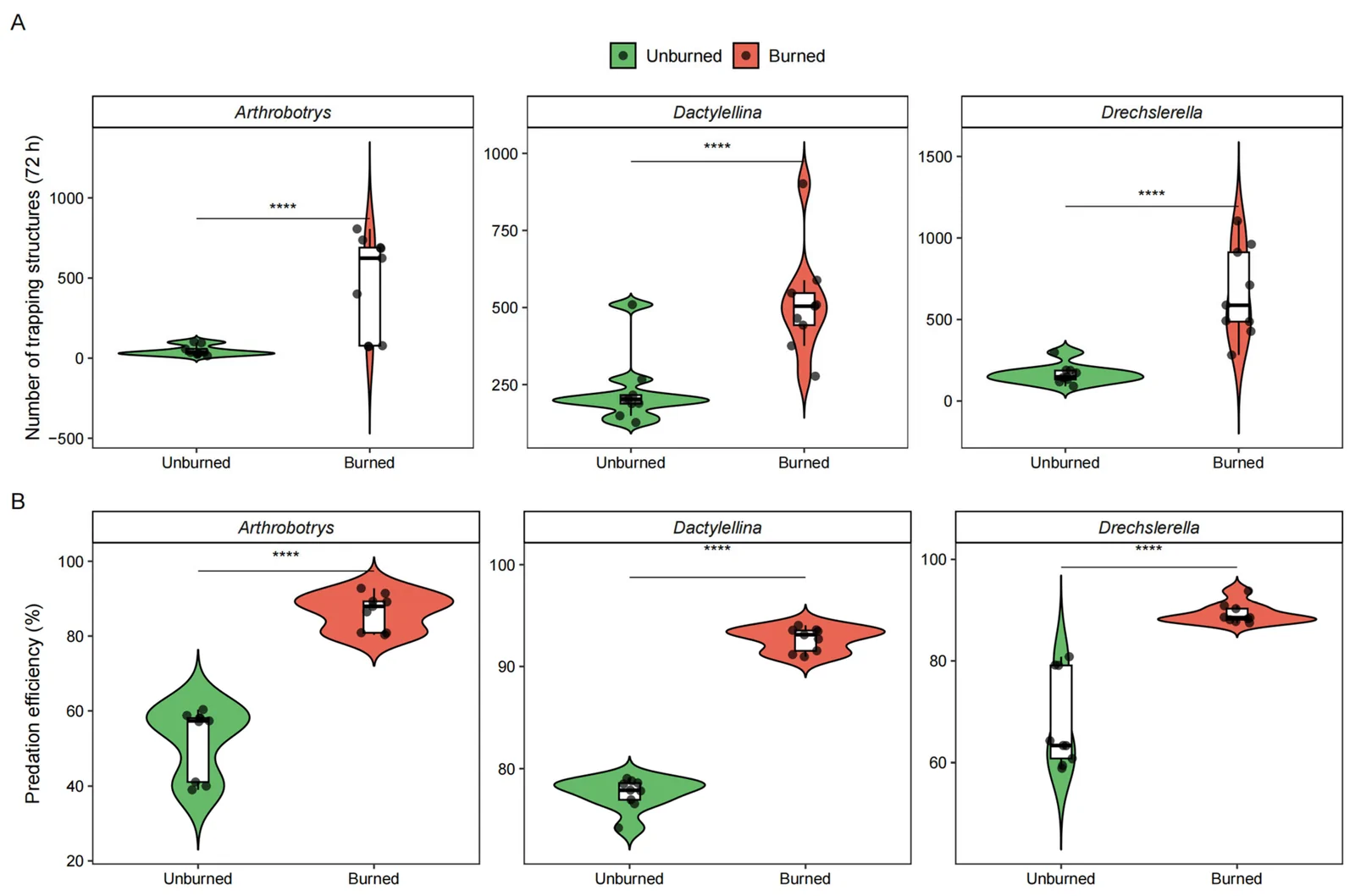
Trapping-structure production within 72 h (**A**) and corrected predation efficiency (**B**) of nematode-trapping fungi isolated from naturally burned and adjacent unburned habitats. Violin plots show strain-level means for the three genera (*Arthrobotrys*, *Dactylellina*, and *Drechslerella*). Green and red indicate strains from unburned and burned habitats, respectively. Asterisks indicate significant differences between habitat origins within each genus based on Tukey-adjusted mixed-model contrasts; ****: *p* < 0.0001.

Across all three genera, burned-habitat strains produced more trapping structures and had higher predation efficiency than unburned strains (all Tukey-adjusted *p* < 0.0001; Figure 3, Table 3). The response was strongest in *Arthrobotrys*, intermediate in *Drechslerella*, and weakest in *Dactylellina*.

### 3.2. Vertical Redistribution Experiment Results

We tracked occurrence dynamics within stratified artificial soil microcosms following simulated fire. Across treatments, fire generated a strong vertical thermal gradient and markedly altered the spatial distribution of NTF along the soil profile. The topsoil-associated species *A. oligospora* was rapidly lost after fire, whereas the deep-soil species *Dac. ellipsospora* and *Dre. dactyloides* were progressively detected in upper layers, indicating upward redistribution.

#### 3.2.1. Thermal Gradient Generated by Simulated Fire

Simulated fire produced a steep temperature gradient with depth (Supplementary Figure S7). Maximum temperature reached 474 °C at 2 cm, 84.1 °C at 5 cm, 58.8 °C at 10 cm, 44.5 °C at 15 cm, and 37.5 °C at 20 cm. Heating was therefore concentrated in the upper soil, whereas deeper layers experienced substantially weaker thermal stress. This pattern suggests that fire created strong vertical environmental heterogeneity, with severe heating in the topsoil but relative thermal buffering at depth in this experimental system.

#### 3.2.2. Vertical Redistribution of NTF After Fire

Time-depth occurrence patterns showed clear post-fire redistribution of NTF across soil layers.

**Figure 4:**
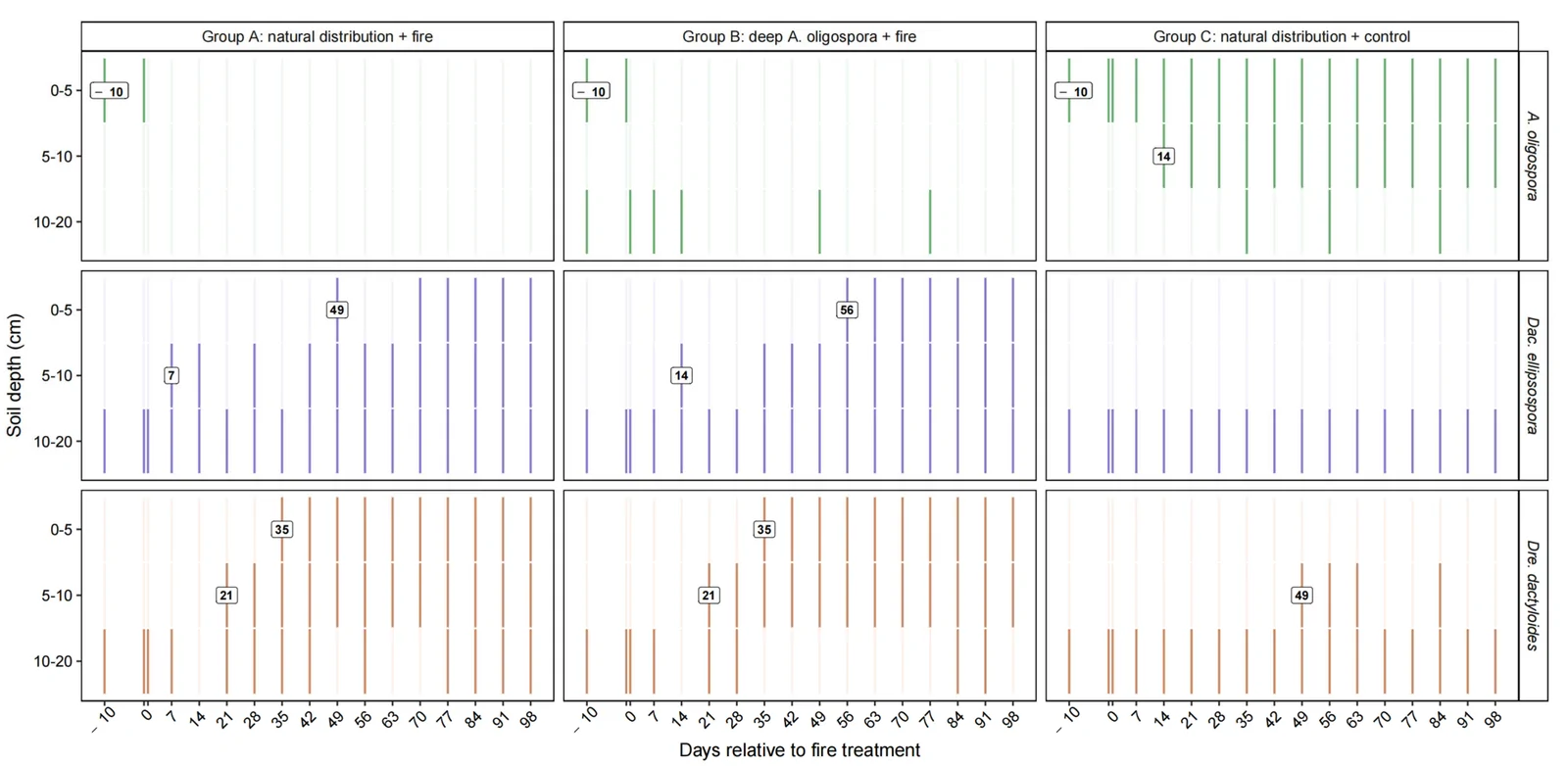
Vertical redistribution of nematode-trapping fungi across soil profiles following simulated fire. Tiles indicate detection status over time and across depth for *A. oligospora*, *Dac. ellipsospora* and *Dre. dactyloides* in the three experimental groups. Darker cells indicate detection (≥1 of 9 bait plates positive) and lighter cells indicate non-detection (0/9 plates). Numbers mark the first sampling day on which each species was detected in the 5–10 cm and 0–5 cm layers. The number of positive plates out of nine is given in Supplementary Table S1 positive plates.

In Group A, *A. oligospora* was restricted to the 0–5 cm before fire, whereas *Dac. ellipsospora* and *Dre. dactyloides* occurred only in deep soil before treatment. After fire, *A. oligospora* was no longer detected in the topsoil throughout the experiment. By contrast, *Dac. ellipsospora* first appeared in the 5–10 cm layer on day 7 and in the 0–5 cm layer on day 49, whereas *Dre. dactyloides* first appeared in the 5–10 cm and 0–5 cm layers on days 21 and 35, respectively. Thus, both deep-soil taxa showed a stepwise upward redistribution from deep soil to the intermediate and then to the topsoil (Figure 4, Group A).

In Group B, *A. oligospora* was present in both the 0–5 cm and 10–20 cm layers before fire. After fire, topsoil *A. oligospora* was again eliminated and never recovered, although it remained detectable at some time points in deep soil. In contrast, *Dac. ellipsospora* first entered the 5–10 cm layer on day 14 and the 0–5 cm layer on day 56, whereas *Dre. dactyloides* reached the 5–10 cm and 0–5 cm layers on days 21 and 35, respectively. Deep-soil supplementation of *A. oligospora* therefore did not restore its topsoil population, whereas both deep-soil taxa exhibited clear patterns of upward redistribution (Figure 4, Group B).

In the unburned control (Group C), *A. oligospora* remained stable in the 0–5 cm layer throughout the experiment and appeared in the 5–10 cm layer from day 14 onward, with occasional detections in the deep layer later in the experiment. By contrast, *Dac. ellipsospora* remained confined to the 10–20 cm layer throughout, and *Dre. dactyloides* was detected primarily in the 10–20 cm layer, with only occasional late detections in 5–10 cm layer and no stable occurrence in the topsoil. Thus, vertical stratification remained largely intact in the absence of fire, whereas fire was associated with upward redistribution of deep-soil taxa (Figure 4, Group C). The contrasting patterns between Group C and the fire treatments suggest that the presence of *A. oligospora* in the topsoil may have competitively excluded the deep-soil taxa from upper layers under unburned conditions, but this hypothesis requires further testing.

#### 3.2.3. Qualitative Recolonization Metrics

First-detection timing further highlighted consistent differences among taxa. *Dac. ellipsospora* reached the 5–10 cm layer earlier than *Dre. dactyloides* in Group A (day 7 vs. day 21) and Group B (day 14 vs. day 21), whereas both species eventually reached the topsoil under fire treatment. In contrast, *A. oligospora* never re-established in the 0–5 cm layer in either fire treatment, even when deep-soil inoculum was added in Group B. Together, these bait-based patterns indicate that post-fire topsoil reassembly was not simply determined by the presence of deep-soil propagules, but was associated with taxon-specific vertical distribution capacity. Deep soil therefore acted as a source pool for post-fire recolonization, but only for taxa adapted to post-fire topsoil conditions.

## 4. Discussion

By integrating strain-level functional trait quantification and stratified soil microcosm assays, this study delivers direct experimental evidence that NTF respond to wildfire disturbance via two complementary mechanisms: intraspecific functional trait shifts and community-wide vertical redistribution. Our findings clarify core recovery mechanisms for NTF and establish a general mechanistic framework for how soil microbes cope with abrupt, spatially heterogeneous environmental perturbations.

### 4.1. Post-Fire Trait Reorganization: Functional Shifts Under Resource Limitation

Strains isolated from burned habitats consistently showed smaller conidia, more trapping structures, and higher predation efficiency relative to conspecifics from unburned sites. This pattern is consistent with the reorganization of soil resource conditions after fire. Fire commonly reduces soil nitrogen availability through organic matter combustion and nitrogen volatilization, thereby intensifying nitrogen limitation during the early post-fire period [31,32,33]. Under such conditions, increased predation on nematodes may provide an important compensatory pathway for nitrogen acquisition and post-fire establishment.

Existing laboratory evidence confirms that nitrogen scarcity triggers trap formation and boosts predatory capacity across multiple NTF taxa: Olthof and Estey found that medium carbon and nitrogen concentrations were negatively correlated with the nematophagous activity of *A. oligospora* [34]. Liang et al. further demonstrated that nitrate assimilation pathways regulate trap development [35], while Zhang et al. reported that urea supplementation elevates the predation efficiency of *A. oligospora* by 34.2% [36]. Conversely, severe carbon or nitrogen restriction suppresses both hyphal growth and trap production [37]. Although direct experimental data linking nitrogen availability to conidia size remain scarce for NTF, studies on other fungal groups confirm that sporulation morphology shifts in response to the nutrient composition of the medium.

Nonetheless, from a trait-allocation perspective, the smaller conidia observed in burned-habitat strains could indicate a shift in resource investment away from reproduction and towards resource-acquisition structures, a pattern that would be consistent with the “rapid resource acquisition” strategies reported in other post-fire fungi [38]. However, because we did not measure conidial number, total conidial biomass, sporulation rate, or carbon/nitrogen allocation, this resource-allocation interpretation remains a hypothesis that requires direct testing. Rather than maintaining high reproductive investment, post-fire environments are likely to favour trait combinations that promote rapid exploitation of limited resources [39], stronger predatory performance, and greater hyphal expansion. Our results therefore suggest that the observed trait shifts are consistent with a broader functional reorganization under post-fire resource constraint, highlighting the capacity of soil microorganisms to adjust niche use over short time scales through trait adjustments.

### 4.2. Taxon-Specific Responses: Divergent Pathways Among Life-History Strategies

Responses to fire differed strongly among genera, indicating that post-fire adjustment was strategy dependent. *Arthrobotrys* (more saprotrophic) showed the strongest increase in predatory performance, whereas *Dactylellina* and *Drechslerella* (more predatory) responded primarily through faster hyphal growth. This divergence suggests that NTF do not respond to post-fire environments along a single functional axis, but instead adjust along different trait dimensions. These contrasting responses are consistent with differences in baseline resource-use strategies. Under normal conditions, *Arthrobotrys* relies more heavily on saprotrophic nutrition [40], so post-fire declines in organic resources may force a stronger shift towards predation [25], expressed as increased trapping-structure production and higher predation efficiency. By contrast, *Dactylellina* and *Drechslerella* are already strongly predatory, and their post-fire advantage may lie less in further strengthening predation than in expanding rapidly into newly available or disturbance-released space through faster hyphal spread. In this sense, post-fire NTF responses are better viewed as a divergence between “predation intensification” and “spatial expansion” than as a uniform increase in predation alone. Post-fire community reassembly is therefore not a one-dimensional process, but is shaped by multiple functional pathways.

### 4.3. Vertical Redistribution: Deep Soil as a Spatial Insurance Mechanism

The artificial soil-profile experiment showed that deep-soil populations of *Dac. ellipsospora* and *Dre. dactyloides* were progressively detected in upper soil layers and eventually reached the topsoil, whereas the topsoil-associated species *A. oligospora* was eliminated by fire and did not recover. While our data cannot distinguish active hyphal migration from passive transport or differential survival, and dead or heat-killed inoculum controls were not included, we cannot completely exclude passive transport. The stepwise detection in intermediate layers before topsoil appearance is nevertheless consistent with upward growth or movement through the soil profile. These results indicate that deep soil not only preserves viable source populations after fire, but can also contribute directly to post-disturbance replenishment of damaged surface layers. However, because actual dispersal or growth rates were not measured, the observed differences in detection timing should be interpreted as taxon-specific redistribution patterns rather than demonstrated differences in migration capacity.

This pattern is consistent with the spatial insurance hypothesis, which predicts that spatial heterogeneity can stabilize communities by providing buffered habitats [18,41,42]. In our system, fire generated a strong vertical thermal gradient, with severe heating in the topsoil but substantial thermal buffering at depth [7,43]. Under this vertical heterogeneity, deep-soil source populations persisted and subsequently expanded upward, as evidenced by their detection in shallower layers [15]. Importantly, our results move this idea beyond a conceptual refuge effect by identifying its temporal scale and directionality: deep-soil sources did not merely remain latent, but were detected in upper layers over a period of weeks, suggesting active recolonization. However, because dead-inoculum controls were not used, we cannot fully separate active biological growth from passive transport; future studies incorporating such controls will be necessary to rigorously demonstrate active migration. In addition, because our experiment used sterile soil in enclosed pots, natural processes such as horizontal dispersal, root-mediated transport, and faunal interactions were excluded. Therefore, the observed patterns represent one component of post-fire recovery, and the relative contribution of vertical versus horizontal movement in natural soils remains to be assessed. Despite these limitations, deep soil harbours viable source populations that can contribute to the replenishment of surface layers following fire and should be viewed as a potentially important component of microbial recovery rather than simply a passive refuge.

### 4.4. Joint Mechanisms: An Integrated Framework of Trait Adjustment and Spatial Replenishment

Taken together, our results suggest that NTF responses to fire are jointly shaped by spatial replenishment and functional trait adjustment. Fire removes disturbance-sensitive taxa from topsoil and simultaneously creates new spatial opportunities and resource constraints across the soil profile [7,43]. Deep-soil source populations then determine which taxa can re-enter shallow soil [30], whereas subsequent establishment and expansion depend on whether their functional traits are suited to post-fire conditions [44,45]. In other words, spatial replenishment governs who can arrive, whereas trait adjustment influences who can establish.

Notably, the trait comparison assay and vertical redistribution microcosm experiment used completely independent fungal strain sets. We cannot directly link strain-specific trait phenotypes observed in culture to the recolonization performance detected in soil profiles; the two datasets provide only complementary, indirect evidence for the integrated recovery framework proposed herein, rather than a direct causal linkage between trait shifts and upward soil redistribution.

This perspective suggests that post-fire community reassembly is not the outcome of dispersal or environmental filtering alone, but of their sequential and complementary interaction. The main contribution of this study therefore lies not only in documenting post-fire responses in NTF, but also in proposing and supporting an integrated framework of trait adjustment plus spatial replenishment. More broadly, this framework offers a unified view of how soil microorganisms reorganize under rapid environmental change [43,46].

### 4.5. Limitations and Future Directions

Despite providing new experimental evidence, this study has several limitations. First, the trait contrasts were based on strains isolated from naturally burned versus adjacent unburned habitats. Although the same species were compared across habitats, which controls for species identity, our comparative design cannot distinguish the contributions of phenotypic plasticity, environmental filtering, and local adaptation. Future work should combine common-garden experiments, longer-term culturing, and genomic analyses to disentangle these processes. Second, the artificial soil-profile experiment necessarily simplified many components of natural soil environments, including interactions with plant roots, bacteria, and other soil fauna, and therefore likely underestimates the complexity of post-fire dynamics in situ. The complete failure of *Arthrobotrys* to recolonize the topsoil in our experiment contrasts with its documented presence, albeit at reduced abundance, in naturally burned soils [25], indicating that the enclosed pot design excluded important recolonization pathways such as horizontal dispersal from adjacent unburned areas. In addition, the bait-based detection method captured only culturable, inducible fungi, rather than quantitative in situ abundance. Direct molecular quantification (e.g., qPCR) would complement bait-based detection and provide quantitative information on fungal abundance in future studies. Third, the vertical redistribution component was based on qualitative detection–nondetection criteria. Although this was sufficient to reveal the direction and timing of first appearance, it does not capture changes in population size or colonization intensity. Moreover, dead or heat-killed inoculum controls were not included in the experimental design; therefore, we cannot completely exclude the possibility that passive transport contributed to the observed upward detection of deep-soil taxa. Fourth, the simulated fire, while producing a realistic temperature gradient, was applied to small pots and may not fully replicate the spatial heterogeneity of natural fires.

Future research could extend this framework in at least three directions. First, it will be important to test whether the same combination of trait adjustment and spatial replenishment operates under other forms of disturbance, such as drought, warming or soil management. Second, linking functional trait shifts to genomic features may clarify the molecular basis of rapid microbial responses [47]. Third, from an applied perspective, evaluating how deep-soil structural retention and post-fire soil management influence microbial recovery could help develop more functional-trait-based strategies for the restoration of burned sites. Finally, direct experimental manipulation of fire conditions using genetically identical strains would allow rigorous testing of true phenotypic plasticity.

## 5. Conclusions

This study demonstrates that nematode-trapping fungi exhibit consistent trait differences associated with fire history and that deep-soil taxa can undergo vertical redistribution following simulated fire. These results highlight the potential for coordinated trait shifts and spatial dynamics to contribute to post-disturbance microbial community reassembly. Our findings support a framework in which deep soil serves as a source pool and functional traits influence establishment success, offering a basis for trait-based approaches to understanding and managing post-fire soil ecosystems.

## Figures and Tables

**Table 1:** Spore size and variation range of NTF strains from burned and unburned habitats.

Species	Habitat Type	Length (μm)	Rangeability (μm)	*p* Value	Width (μm)	Rangeability (μm)	*p* Value
*A. oligospora*	Unburned	11.75 ± 0.87	−0.84	0.281	6.80 ± 0.39	−0.12	0.865
	Burned	10.91 ± 0.78			6.68 ± 1.04		
*A. conoides*	Unburned	13.82 ± 1.48	−0.33	0.742	7.12 ± 0.23	−0.58	0.084
	Burned	13.48 ± 0.55			6.55 ± 0.35		
*A. musiformis*	Unburned	16.37 ± 0.84	−2.04	0.048	5.58 ± 0.61	−1.11	0.0084
	Burned	14.33 ± 0.15			4.47 ± 0.57		
*Dac. ellipsospora*	Unburned	43.83 ± 0.66	−4.41	0.006	13.67 ± 0.03	−2.10	0.003
	Burned	39.41 ± 0.10			11.56 ± 0.23		
*Dac. drechsleri*	Unburned	46.01 ± 0.90	−7.89	0.002	13.79 ± 0.22	−2.14	0.055
	Burned	38.12 ± 0.36			11.65 ± 0.97		
*Dac. gephyropaga*	Unburned	49.88 ± 0.78	−8.00	0.002	18.48 ± 0.20	−1.77	<0.001
	Burned	41.88 ± 0.20			16.71 ± 0.12		
*Dre. Dactyloides*	Unburned	20.88 ± 0.76	−1.70	0.226	3.74 ± 0.12	0.46	0.291
	Burned	19.19 ± 1.73			4.20 ± 0.56		
*Dre. aphrobrocha*	Unburned	50.61 ± 0.65	−3.31	0.003	22.95 ± 0.07	−3.35	0.028
	Burned	47.30 ± 0.59			19.59 ± 1.00		
*Dre. coelobrocha*	Unburned	50.23 ± 2.36	−3.55	0.105	21.35 ± 0.29	−2.18	0.003
	Burned	46.68 ± 1.53			19.17 ± 0.41		

Note: Values represent the mean ± SD of strain-level averages (*n* = 3 strains per habitat, each with 300 measured mature conidia). Difference = the mean trait value of burned isolates minus the mean trait value of unburned isolates. *p*-values were derived from independent two-sample *t*-tests of strain means.

**Table 2:** Colony diameters and variation ranges of NTF strains from naturally burned and adjacent unburned habitats.

Species	Habitat Type	Colony Diameter (mm)	Rangeability (mm)	*p* Value
*A. oligospora*	Unburned	8.14 ± 0.43	−0.25	0.046
	Burned	7.89 ± 0.12		
*A. conoides*	Unburned	7.31 ± 0.41	1.31	0.008
	Burned	8.62 ± 0.29		
*A. musiformis*	Unburned	8.15 ± 0.58	−2.95	0.005
	Burned	5.20 ± 0.27		
*Dac. ellipsospora*	Unburned	0.34 ± 0.07	0.82	0.002
	Burned	1.16 ± 0.07		
*Dac. drechsleri*	Unburned	0.47 ± 0.06	1.33	<0.001
	Burned	1.80 ± 0.07		
*Dac. gephyropaga*	Unburned	1.03 ± 0.09	0.66	0.003
	Burned	1.69 ± 0.08		
*Dre. dactyloides*	Unburned	1.51 ± 0.08	4.60	<0.001
	Burned	6.11 ± 0.20		
*Dre. aphrobrocha*	Unburned	0.51 ± 0.07	1.16	0.002
	Burned	1.67 ± 0.13		
*Dre. coelobrocha*	Unburned	0.84 ± 0.05	1.03	0.001
	Burned	1.87 ± 0.10		

Note: Values represent the mean ± SD of strain-level means (*n* = 3 strains per habitat, each based on 5 replicate plates). *p*-values are from independent-samples *t*-tests of strain-level means.

**Table 3:** The changes in the number of tapping structure and predation rates of NTF strains in naturally burned and adjacent unburned habitats.

Species	Habitat Type	Trapping Device (Count)	Rangeability (Count)	*p* Value	Predation Efficiency (%)	Rangeability (%)	*p* Value
*A. oligospora*	Unburned	61.20 ± 37.04	681.60	0.002	59.04 ± 1.20	31.18	<0.001
	Burned	742.80 ± 57.41			90.22 ± 3.04		
*A. conoides*	Unburned	84.07 ± 35.07	487.13	0.008	40.02 ± 1.48	40.65	<0.001
	Burned	571.20 ± 133.55			80.67 ± 0.57		
*A. musiformis*	Unburned	21.36 ± 13.65	53.24	0.014	57.51 ± 1.30	31.28	<0.001
	Burned	74.60 ± 8.99			88.79 ± 1.00		
*Dac. ellipsospora*	Unburned	321.33 ± 144.11	323.00	0.043	77.54 ± 1.24	14.72	0.001
	Burned	644.33 ± 201.20			92.26 ± 1.24		
*Dac. drechsleri*	Unburned	198.67 ± 15.68	321.33	0.003	78.71 ± 1.77	14.81	0.002
	Burned	520.00 ± 27.12			93.52 ± 0.46		
*Dac. gephyropaga*	Unburned	163.93 ± 44.33	208.67	0.025	76.50 ± 2.09	15.75	0.004
	Burned	372.60 ± 80.28			92.25 ± 1.61		
*Dre. dactyloides*	Unburned	205.57 ± 74.81	289.43	0.048	63.63 ± 1.34	26.53	<0.001
	Burned	495.00 ± 181.09			90.16 ± 2.86		
*Dre. aphrobrocha*	Unburned	116.87 ± 22.96	383.53	0.004	79.70 ± 1.57	9.17	0.012
	Burned	500.40 ± 79.90			88.87 ± 2.91		
*Dre. coelobrocha*	Unburned	169.80 ± 24.07	823.33	<0.001	59.71 ± 1.73	29.15	<0.001
	Burned	993.13 ± 96.46			88.86 ± 1.52		

Note: Trap counts and predation efficiency were measured using five replicate observation chambers per strain; values represent the strain-level mean ± SD. *p*-values were derived from independent two-sample *t*-tests of strain averages.

## Data Availability

The raw data supporting the conclusions of this article will be made available by the authors on request.

## Supplementary Materials

The following supporting information can be downloaded at https://www.mdpi.com/article/10.3390/jof12080611/s1. Supplementary Table S1: NTF strains from natural burned and unburned habitat and used in the trait differences experiment. Supplementary Table S2: Fixed-effect coefficients from linear mixed-effects models (LMMs) and generalized linear mixed-effects models (GLMMs) for conidial size, hyphal growth rate, trapping-structure production, and predation efficiency. Supplementary Table S3: Tukey adjusted pairwise contrasts between burned and unburned habitats within each genus for all measured traits. Supplementary Figure S1: Schematic diagrams illustrating conidial measurements (A, B) and the measurement of hyphal growth rate in NTF (C). Supplementary Figure S2: Assistive device used to measure predation efficiency. Right: CMA plate with spore inoculation wells and observation chambers. Left: the assistive device. Supplementary Figure S3: Conidia and trap structure of nematode-trapping fungi strains used in the migration experiment. (A) *Arthrobotrys oligospora*; (B) *Dactylellina ellipsospora*; (C) *Drechslerella dactyloides*. Supplementary Figure S4: Schematic diagram of the preparation of NTF plugs. Supplementary Figure S5: Schematic diagram of NTF community construction. Species are represented by their corresponding conidial shapes. Supplementary Figure S6: Photograph of the experimental quadrats used in the migration experiment. Supplementary Figure S7: Temperaturedynamics at different soil depths during the simulated fire treatment. Thermocouples were placed at depths of 2, 5, 10, 15, and 20 cm in a blank pot filled with sterile soil and heated under identical conditions. Temperatures was recorded every 10 min from the start of heating until values returned to pre-treatment levels.
