# Supplementary material for: Trait Shifts and Vertical Redistribution Enable Nematode-Trapping Fungi to Adapt to Fire Disturbance
Source: J Fungi (Basel). 2026 Aug 14;12(8):611. doi: 10.3390/jof12080611 (PMC13514460; doi:10.3390/jof12080611)
Supplement: Supplementary file 1 [file jof-12-00611-s001.zip › jof-4410408-supplementary.pdf]

Table. S1. NTF strains from natural burned and unburned habitat and used in the trait differences experiment.

Table S2. Fixed-effect coefficients from linear mixed-effects models (LMMs) and generalized linear mixed-effects models (GLMMs) for conidial size, hyphal growth rate, trapping-structure production, and predation efficiency.

Table S3. Tukey-adjusted pairwise contrasts between burned and unburned habitats within each genus for all measured traits.

Fig. S1. Schematic diagrams illustrating conidial measurements (A, B) and the measurement of hyphal growth rate in NTF (C).

Fig. S2. Assistive device used to measure predation efficiency. Right: CMA plate with spore inoculation wells and observation chambers. Left: the assistive device.

Fig. S3. Conidia and trap structure of nematode-trapping fungi strains used in the migration experiment. (A) *Arthrobotrys oligospora*; (B) *Dactylellina ellipsospora*; (C)*Drechslerella dactyloides*.

Fig. S4. Schematic diagram of the preparation of NTF plugs.

Fig. S5. Schematic diagram of NTF community construction. Species are represented by their corresponding conidial shapes.

Fig. S6. Photograph of the experimental quadrats used in the migration experiment.

Fig. S7. Temperaturedynamics at different soil depths during the simulated fire treatment. Thermocouples were placed at depths of 2, 5, 10, 15, and 20 cm in a blank pot filled with sterile soil and heated under identical conditions. Temperatures was recorded every 10 min from the start of heating until values returned to pre-treatment levels.

Table. S1.

Genus	Species	Strain Code<br>Strains from burned habitat	 Strains from adjacent unburned habitat	Note<br>（conidial type）
		XJ049	DL2	
	A. oligospora	XJ048	DL219	
		ZX111	DL252	
		XJ001	DL3	
Arthrobotrys	*A. musiformis*	XJ019	DL6	Arthrobotrys-shaped
		WQ40	DL40	
		YS04	DL169	
	A. conoides	YS05	DL191	
		YS06	DL222	
		XJ424	WQ53	
	Dac. drechsleri	XJ145	XJ127	
		GD142	XJ142	
		XJ40	DL251	
Dactylellina	*Dac. ellipsospora*	XJ130	TB72	Monacrosporium-shaped
		XJ209	LP433	
		XJ10	LP444	
	Dac. gephyropaga	XJ399	XJ50	
		XJ60	XJ58	
		YS08	LP155	
	Dre. dactyloides	YS11	LP352	Arthrobotrys-shaped
		YS12	LP381	
		XJ003	LP313	
Drechslerella	*Dre. aphrobroha*	XJ025	XJ85	
		XJ109	XJ63	Monacrosporium-shaped
		XJ120	DL126	
	Dre. coelobrocha	XJ007	DL90	
		XJ69	DL106	

Table. S2.

model	term	estimate	std.error	statistic	df	p.value
Conidia length	(Intercept)	13.9794	5.6204	2.4873	6.028	0.0472
Conidia length	Habitat Burned	-1.0713	0.5422	-1.976	42	0.0548
Conidia length	Genus *Dactylellina*	32.5916	7.9485	4.1003	6.028	0.0063
Conidia length	Genus *Drechslerella*	26.5951	7.9485	3.3459	6.028	0.0154
Conidia length	Habitat Burned:genus *Dactylellina*	-5.6952	0.7667	-7.428	42	0.0000
Conidia length	Habitat Burned:genus *Drechslerella*	-1.7819	0.7667	-2.3241	42	0.025
Conidia width	(Intercept)	6.5022	3.3942	1.9157	6.0303	0.1036
Conidia width	Habitat Burned	-0.6016	0.3403	-1.7677	42	0.0844
Conidia width	Genus *Dactylellina*	8.8121	4.8002	1.8358	6.0303	0.1158
Conidia width	Genus *Drechslerella*	9.5115	4.8002	1.9815	6.0303	0.0946
Conidia width	Habitat Burned:genus *Dactylellina*	-1.4034	0.4813	-2.916	42	0.0057
Conidia width	Habitat Burned:genus *Drechslerella*	-1.0916	0.4813	-2.2681	42	0.0285
Hyphal growth rate	(Intercept)	7.8689	0.5759	13.6637	6.2367	0.0000
Hyphal growth rate	Habitat Burned	-0.6311	0.1594	-3.9589	258	0.0001
Hyphal growth rate	Genus *Dactylellina*	-7.2589	0.8144	-8.9127	6.2367	0.0001
Hyphal growth rate	Genus *Drechslerella*	-6.9167	0.8144	-8.4925	6.2367	0.0001
Hyphal growth rate	Habitat Burned:genus *Dactylellina*	1.5733	0.2254	6.9787	258	0.0000
Hyphal growth rate	Habitat Burned:genus *Drechslerella*	2.8989	0.2254	12.8583	258	0.0000
Trap number	(Intercept)	3.6983	0.2827	13.0805	NA	0.0000
Trap number	Habitat Burned	2.1175	0.0666	31.7707	NA	0.0000
Trap number	Genus *Dactylellina*	1.6593	0.399	4.159	NA	0.0000
Trap number	Genus *Drechslerella*	1.3742	0.399	3.4437	NA	0.0006
Trap number	Habitat Burned:genus *Dactylellina*	-1.2781	0.0892	-14.3296	NA	0.0000
Trap number	Habitat Burned:genus *Drechslerella*	-0.7473	0.0901	-8.2953	NA	0.0000
Predation efficiency (logit)	(Intercept)	0.0879	0.1704	0.5159	6.1812	0.6239
Predation efficiency (logit)	Habitat Burned	1.8345	0.0414	44.2903	240	0.0000
Predation efficiency (logit)	Genus *Dactylellina*	1.1569	0.241	4.8002	6.1812	0.0028
Predation efficiency (logit)	Genus *Drechslerella*	0.6866	0.241	2.8489	6.1812	0.0283
Predation efficiency (logit)	Habitat Burned:genus *Dactylellina*	-0.5261	0.0586	-8.9809	240	0.0000
Predation efficiency (logit)	Habitat Burned:genus *Drechslerella*	-0.4585	0.0586	-7.8269	240	0.0000

Notes: The coefficient habitatBurned represents the effect of fire (Burned - Unburned) for the reference genus *Arthrobotrys*. Interaction terms indicate how the fire effect differs for *Dactylellina* and *Drechslerella* relative to *Arthrobotrys*. For the LMMs (conidia length, conidia width, hyphal growth rate, predation efficiency), denominator degrees of freedom were approximated using the Satterthwaite method. The trap number model was fitted with a negative-binomial GLMM (glmmTMB), which reports Wald z-statistics without denominator degrees of freedom. All P-values are

two-sided.

Table. S3.

model	genus	contrast	estimate	SE	df	statistic	p.value
Conidia length	*Arthrobotrys*	Unburned - Burned	1.0713	0.5422	42	1.976	0.0548
Conidia length	*Dactylellina*	Unburned - Burned	6.7665	0.5422	42	12.4807	0.0000
Conidia length	*Drechslerella*	Unburned - Burned	2.8532	0.5422	42	5.2627	0.0000
Conidia width	*Arthrobotrys*	Unburned - Burned	0.6016	0.3403	42	1.7677	0.0844
Conidia width	*Dactylellina*	Unburned - Burned	2.0049	0.3403	42	5.8915	0.0000
Conidia width	*Drechslerella*	Unburned - Burned	1.6931	0.3403	42	4.9753	0.0000
Hyphal growth rate	*Arthrobotrys*	Unburned - Burned	0.6311	0.1594	240	3.9589	0.0001
Hyphal growth rate	*Dactylellina*	Unburned - Burned	-0.9422	0.1594	240	-5.9105	0.0000
Hyphal growth rate	*Drechslerella*	Unburned - Burned	-2.2678	0.1594	240	-14.2255	0.0000
Trap number	*Arthrobotrys*	Unburned / Burned	0.1203	0.008	Inf	-31.7707	0.0000
Trap number	*Dactylellina*	Unburned / Burned	0.432	0.0256	Inf	-14.151	0.0000
Trap number	*Drechslerella*	Unburned / Burned	0.2541	0.0154	Inf	-22.6033	0.0000
Predation efficiency (logit)	*Arthrobotrys*	Unburned - Burned	-1.8345	0.0414	240	-44.2903	0.0000
Predation efficiency (logit)	*Dactylellina*	Unburned - Burned	-1.3085	0.0414	240	-31.5894	0.0000
Predation efficiency (logit)	*Drechslerella*	Unburned - Burned	-1.3761	0.0414	240	-33.2214	0.0000

Notes: Contrasts were derived from estimated marginal means (emmeans) with Tukey's honestly significant difference adjustment for multiple comparisons. For all traits except trap number, the contrast is Unburned - Burned; positive values indicate larger trait values in unburned than in burned habitats. For trap number (negative binomial GLMM), contrasts are expressed on the response scale as ratios (Unburned / Burned); a ratio < 1 indicates higher trap production in burned than in unburned habitats. The trap number model uses Wald z-statistics (df = Inf); all other models use t-statistics with Satterthwaite-approximated degrees of freedom. P-values reported as 0.0000 indicate P < 0.0001.

Fig. S1.

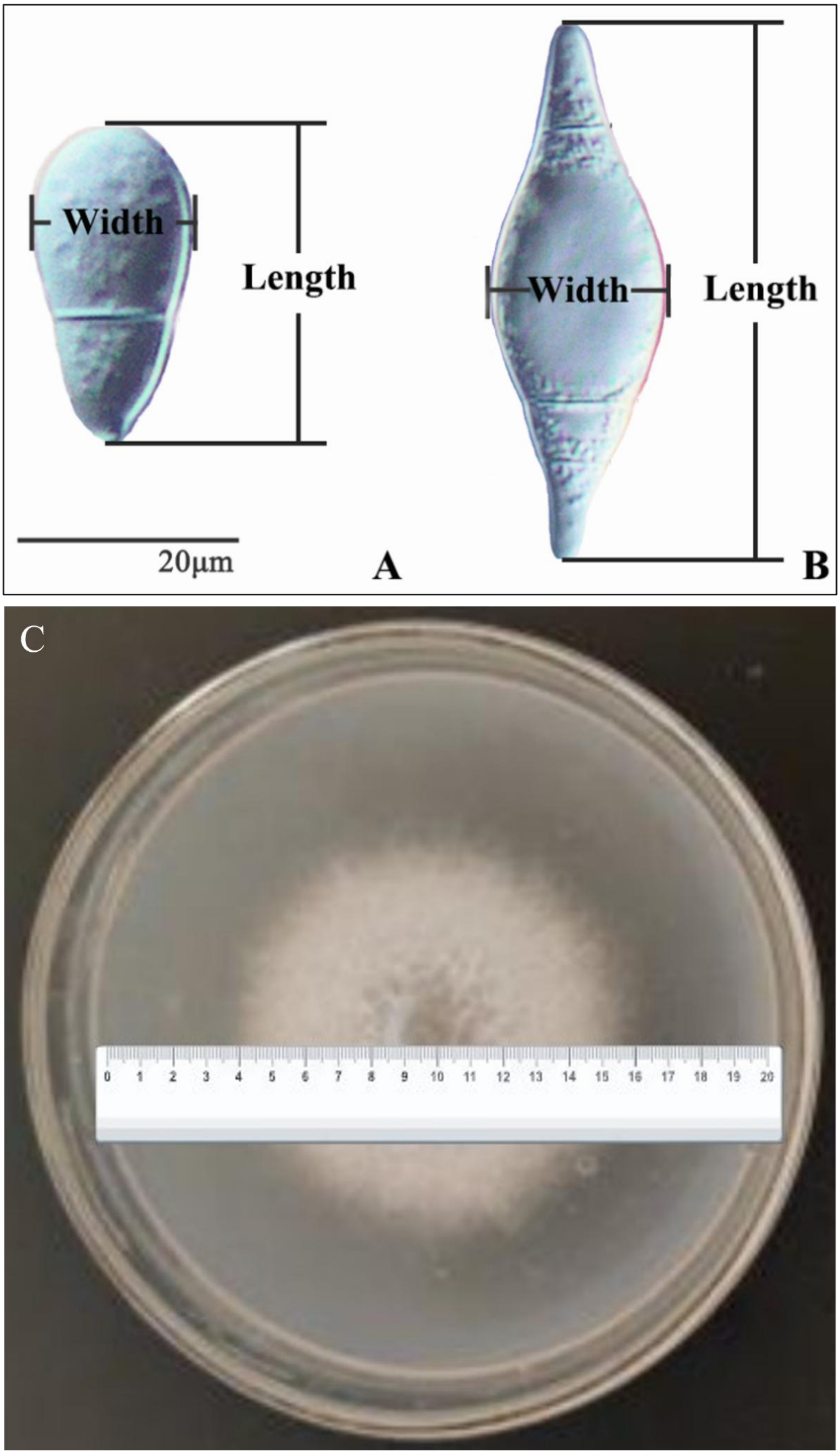
Width
Length
20μm
A
Width
Length
B
C
0 1 2 3 4 5 6 7 8 9 10 11 12 13 14 15 16 17 18 19 20

Fig. S2.

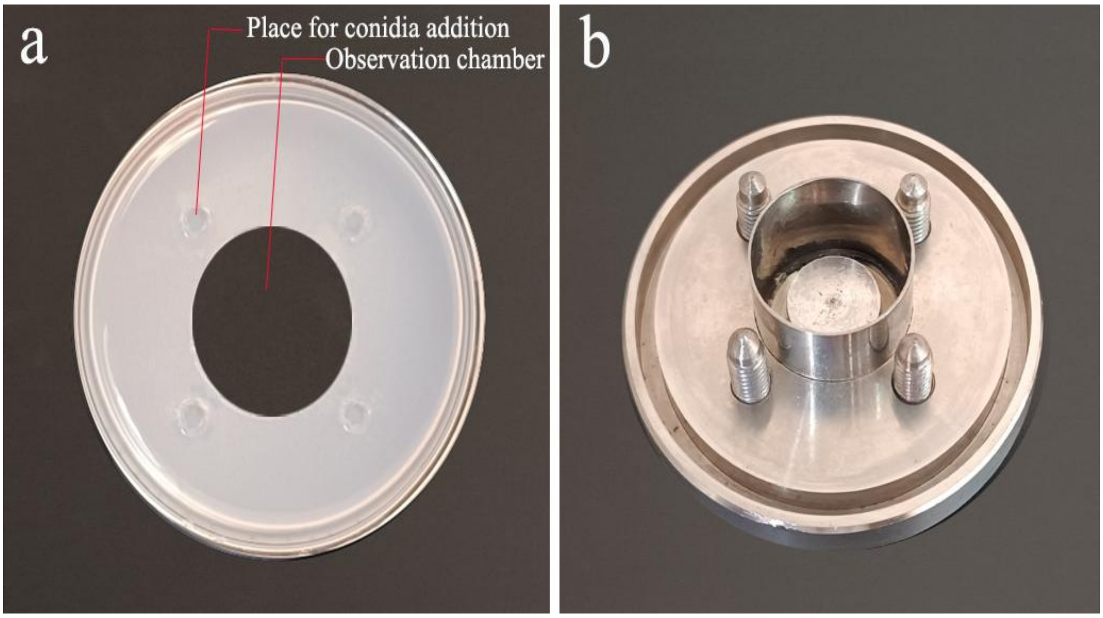

Fig. S3.

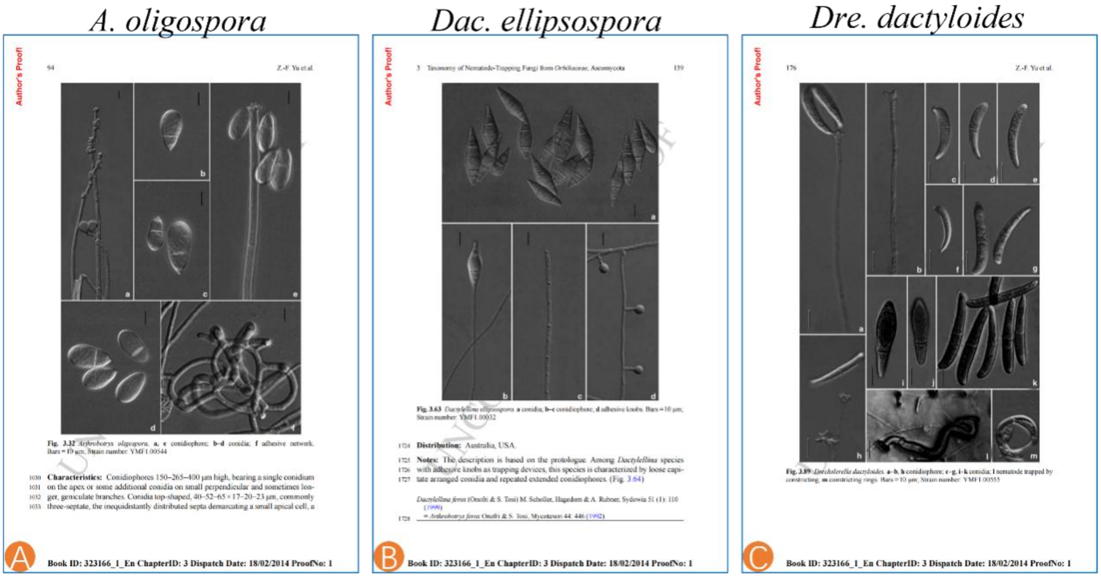

Note:

Panel A. *Arthrobotrys oligospora*: a, conidiophore; b-d, conidia; f, adhesive network (trapping device). Scale bar = 10 µm. (Source: Fig. 3.32, p. 94, Nematode-trapping fungi)

Panel B. *Dactylellina ellipsospora*: a, conidia; b-c, conidiophore; d, adhesive knob (trapping device). Scale bar = 10 µm. (Source: Fig. 3.63, p. 139, Nematode-trapping fungi)

Panel C. *Drechslerella dactyloides*: a-h, conidiophores; c-g, i-k, conidia; m, constricting ring (trapping device) capturing a nematode. Scale bar = 10 µm. (Source: Fig. 3.69, p. 176,

Nematode-trapping fungi)

Fig. S4.

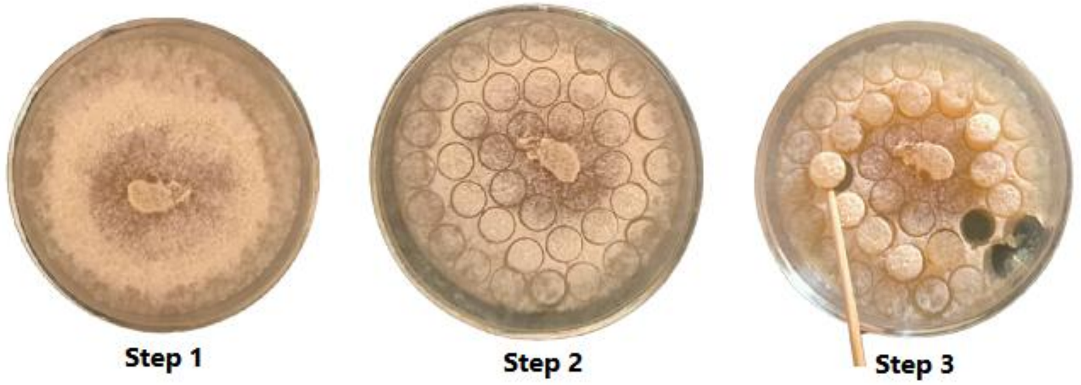

Fig. S5.

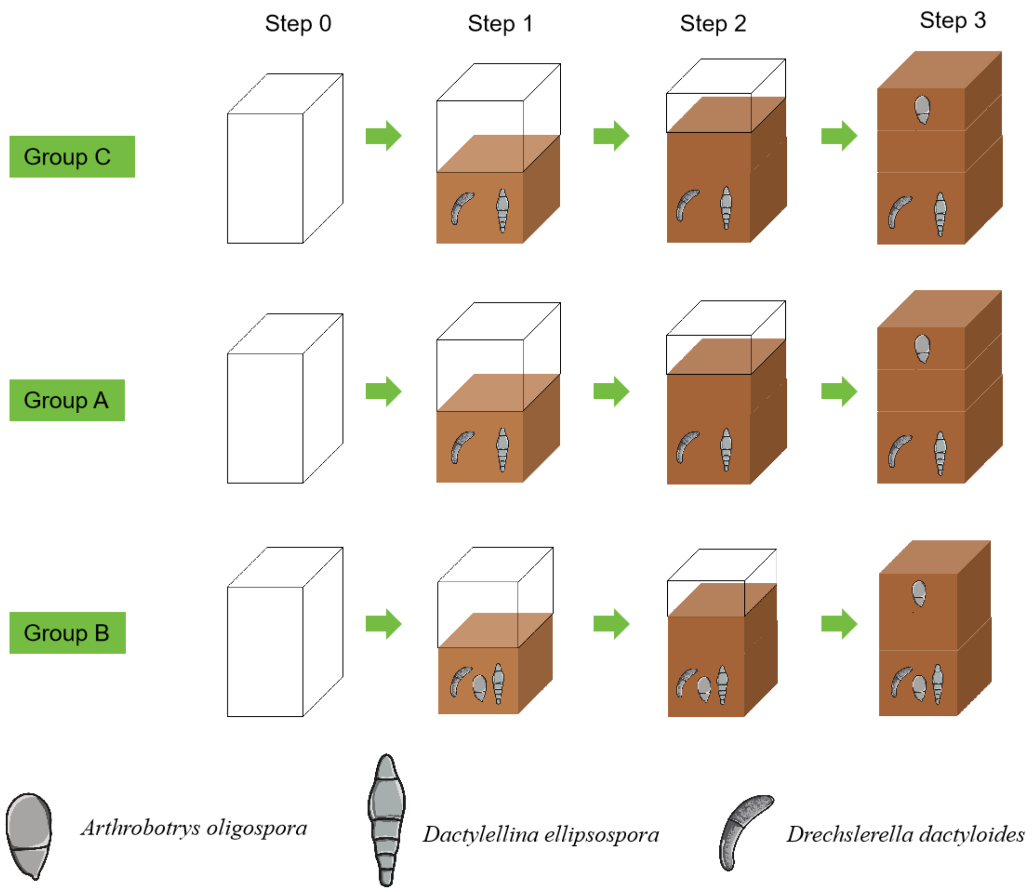

Fig. S6.

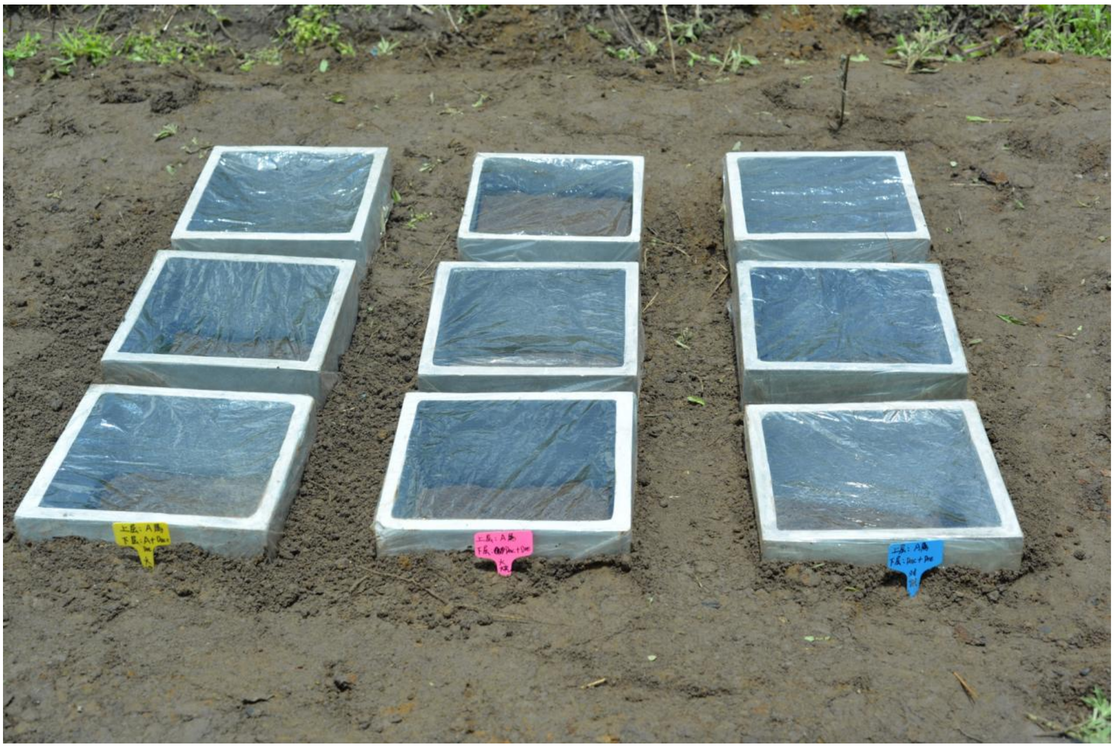

Fig. S7.

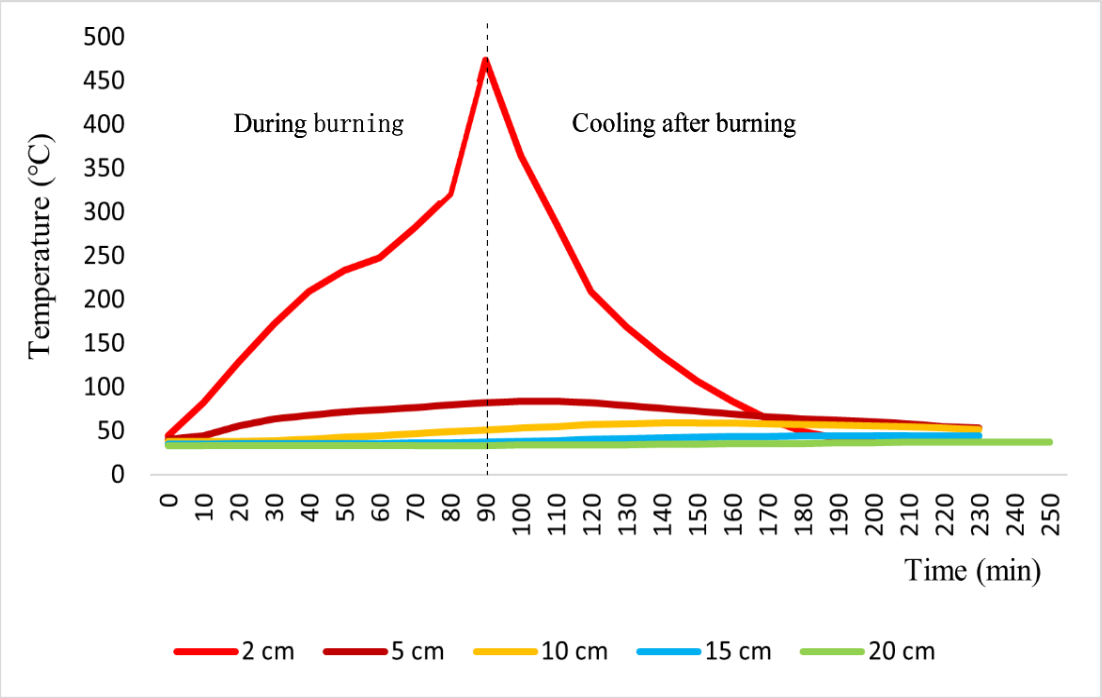
Temperature (°C)
500
450
400
350
300
250
200
150
100
50
0
During burning
Cooling after burning
0
10
20
30
40
50
60
70
80
90
100
110
120
130
140
150
160
170
180
190
200
210
220
230
240
250
Time (min)
2 cm
5 cm
10 cm
15 cm
20 cm

Note: Temperature-time curves adapted from She et al. [7]. Int. J. Wildland Fire 2024, *33*, WF23095. https://doi.org/10.1071/WF23095.
